# A specific blend of prebiotics and postbiotics improved the gut microbiome of dogs with soft stools in the in vitro Simulator of the Canine Intestinal Microbial Ecosystem

**DOI:** 10.1093/jas/skaf056

**Published:** 2025-02-27

**Authors:** Cindy Duysburgh, Celine Nicolas, Mattia Van den Broeck, Fanny Lloret, Patricia Monginoux, Christophe Rème, Massimo Marzorati

**Affiliations:** ProDigest, 9052 Zwijnaarde, Belgium; Virbac SA, 06511 Carros, France; ProDigest, 9052 Zwijnaarde, Belgium; Virbac SA, 06511 Carros, France; Virbac SA, 06511 Carros, France; Virbac SA, 06511 Carros, France; ProDigest, 9052 Zwijnaarde, Belgium; CMET (Center for Microbial Ecology and Technology), University of Ghent, Coupure Links 653, 9000 Ghent, Belgium

**Keywords:** dog, in vitro simulation, intestinal health, Simulator of the Canine Intestinal Microbial Ecosystem

## Abstract

The Simulator of the Canine Intestinal Microbial Ecosystem (SCIME) allows for the study of the long-term effects of food, supplements, or ingredients on the canine gut microbiome in a simulated proximal and distal colon. This model has been used to evaluate the impact of repeated administration of a test product blend composed of a mixture of baobab fruit pulp, acacia gum, heat-killed *Lactobacillus helveticus* HA-122, and specific fractions of selected inactivated yeast strains (including Sacchar*omyces cerevisiae* AQP 12260 and AQP 12988 and *Cyberlindnera jadinii* AQP 12549), on the activity and composition of the gut microbiome of canine donors with soft stools. The SCIME colonic reactors were inoculated with fecal material from 3 different canine donors. After 2 d of stabilization, the 8-d parallel control/treatment period was initiated; reactors were fed with SCIME nutritional medium with or without test product. Changes in microbial metabolic activity were assessed by measuring levels of acetate, propionate, butyrate, lactate, branched short-chain fatty acids, and ammonium. Changes in microbial community composition were assessed using 16S-targeted Illumina sequencing. Overall, test product supplementation resulted in increased saccharolytic fermentation, as evidenced by increases in the health-promoting bacterial metabolites such as propionate (donor-dependent), acetate, and butyrate (donor-dependent) as well as increased abundances of several saccharolytic fermenting microbes, including *Bifidobacterium*. Conversely, proteolytic bacteria like Proteobacteria were reduced with the test product compared to control. Repeated supplementation with the test product was therefore able to induce—in vitro—a positive modulation of the microbiome originated from dogs with soft stools.

## Teaser

This is the first report of the use of the Simulator of the Canine Intestinal Microbial Ecosystem (SCIME) model inoculated with fecal material from canine donors with soft stools. Repeated administration of a test product mixture containing baobab fruit pulp, acacia gum, heat-killed *Lactobacillus helveticus* HA-122, and specific fractions of selected inactivated yeast strains (including *Saccharomyces cerevisiae* AQP 12260 and AQP 12988 and *Cyberlindnera jadinii* AQP 12549) led to a positive modulation of the microbiome in this model, demonstrating increased saccharolytic fermentation and increased abundances of saccharolytic fermenting microbes compared to the control.

## Graphical Abstract

**Graphical Abstract:**
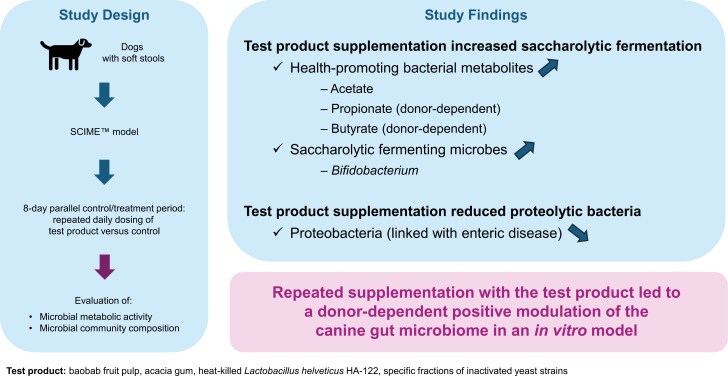

## Introduction

The gut microbiome is a complex collection of microorganisms that is mostly composed of bacteria (Suchodolski, 2011). These bacteria perform several important digestive functions, including the fermentation of non-digestible dietary fibers (Makki et al., 2018). Other important roles of the gut microbiome include providing protection against pathogens, producing important health-related metabolites, and aiding in the development of a healthy immune system (Suchodolski, 2011). Through these activities, a healthy gut microbiome supports the health of its host. Bacteria in the canine gastrointestinal tract generally belong to 1 of the 5 phyla: Firmicutes, Fusobacterium, Bacteroidetes, Proteobacteria, and Actinobacteria (Suchodolski et al., 2008; Honneffer et al., 2017). Firmicutes, Fusobacterium, and Bacteroidetes are the predominant phyla in the fecal microbiome of healthy dogs (Middelbos et al., 2010; Hand et al., 2013). It has been reported that the fecal microbiome of dogs with acute and chronic gastrointestinal diseases is substantially altered relative to that of healthy dogs (Suchodolski et al., 2012), resulting in dysbiosis which encompasses alterations in microbiome diversity and/or structure and functional changes, such as adjustments in microbial metabolite production. A physiological outcome is the production of soft or diarrheic stools.

As mentioned, fermentation of dietary fibers or carbohydrates, termed saccharolytic fermentation, is an important function of the gut microbiome. Short-chain fatty acids (SCFAs) are key byproducts of microbial fermentation that have health-promoting effects. The most abundantly produced SCFAs are acetate, propionate, and butyrate (Sunvold et al., 1995). These metabolites are utilized as an energy source for intestinal epithelial cells, have anti-inflammatory properties, and are involved in the regulation of intestinal motility (Arpaia et al., 2013; Morrison and Preston, 2016; Priyadarshini et al., 2018). SCFAs are produced by some bacteria that are commonly present in the gut microbiome. Many members of the Firmicutes phylum are SCFA producers, suggesting their importance in gut health (Deleu et al., 2021). Lactate is another important byproduct of bacterial fermentation. The gut microbiome includes both lactate-producing and lactate-utilizing bacteria. Lactate produced by lactic acid bacteria can lower the pH of the gut, which has an antimicrobial effect (Wang et al., 2020). Additionally, lactate can be converted to butyrate and/or propionate by lactate-utilizing bacterial species, including members of the Firmicutes phylum, in a process termed cross-feeding (Wang et al., 2020). Following carbohydrate depletion, proteins are fermented by the gut microbiota, resulting in the production of branched SCFAs (bSCFAs) and ammonium along with other compounds such as p-cresol and phenols, with the latter being associated with direct and indirect adverse health effects (Gozdzik et al., 2023).

Prebiotics and probiotics have been shown to improve clinical signs of gastrointestinal disease, including diarrhea. Prebiotics and probiotics, alone or in combination, can help reestablish beneficial bacteria in the canine gut and restore SCFA production (Pilla and Suchodolski, 2021). This study evaluated a specific blend containing 2 prebiotics (baobab fruit pulp and acacia gum) and 2 types of postbiotics (heat-inactivated *Lactobacillus helveticus* HA-122 and fractions of selected inactivated yeast strains including *Saccharomyces cerevisiae* AQP 12260 and AQP 12988 and *Cyberlindnera jadinii* AQP 12549). Both baobab fruit pulp and acacia gum have been shown to have prebiotic properties (Calame et al., 2008; Foltz et al., 2021). Heat-killed whole-cell bacteria or yeast, as well as their derivatives (cell walls and cell extracts), are nowadays considered postbiotics, defined as “a preparation of inanimate microorganisms and/or their components that confers a health benefit on the host” (Salminen et al., 2021), exerting similar effects as probiotics, conferring protection against enteropathogens, demonstrating immunomodulatory effects, and supporting the integrity of the intestinal barrier (Pique et al., 2019). Heat-killed *L. helveticus* HA-122 in particular (formerly *Lactobacillus acidophilus* HA 122) has demonstrated immunomodulatory effects (in vitro and in vivo) and beneficial effects on the gut barrier and digestive signs in different species, including humans (Taverniti and Guglielmetti, 2012; Martinelli et al., 2017; Rawling et al., 2023). Yeast derivatives are common dietary supplements for animals and are also considered postbiotics (Thorakkattu et al., 2022). Supplementation has been associated with several health effects, including immunomodulatory effects, improved intestinal health, and enhanced production of SCFAs (Xue et al., 2017; Lee et al., 2021). The yeast strain derivatives used in the test compound are isolated from selected strains (*S. cerevisiae* AQP 12260, *S. cerevisiae* AQP 12988, and *C. jadinii* AQP 12549). They have been selected by atomic force microscopy and single-molecule force spectroscopy for their capacity to bind microbial pathogens (Schiavone et al., 2014), their immunomodulatory properties, and their beneficial effects on the digestive system and animal growth (Guan et al., 2017; Rawling et al., 2019, 2021).

The Simulator of the Canine Intestinal Microbial Ecosystem (SCIME) is a semi-continuous gastrointestinal tract model that has been validated using a simultaneous in vivo–in vitro comparison (Duysburgh et al., 2020). The SCIME can be utilized to study the long-term effects of food products, supplements, or drugs on the colon-associated microbial community of dogs.

The aim of the study was to assess the impact of repeated administration of a test product composed of a specific blend of baobab fruit pulp, acacia gum, heat-killed *L. helveticus*, and selected yeast strain derivatives on the activity and composition of the gut microbiome of canine donors with soft stools. This study utilized the SCIME model and included 3 canine donors to allow for the assessment of interindividual variability.

## Materials and Methods

### Canine donors and fecal samples

Fecal samples were collected from 3 canine donors with moderate digestive problems and soft stools without the presence of parasites. Samples were collected in sampling boxes and, immediately after collection, an Anaerogen bag was added to the box, which was then sealed to remove all oxygen. Anaerobic phosphate buffered saline was added to the sample and a fecal slurry was prepared following homogenization using a stomacher. Large particles were removed by brief centrifugation (2 min at 500 *g*) and an equal volume of cryoprotectant solution (modified from Hoefman et al. [Hoefman et al., 2013]) was added under anaerobic conditions. Samples were snap-frozen in liquid nitrogen, stored at –80 °C and used within a week. Reactors were inoculated with 5% v/v of a 20% w/v fecal slurry.

### Test product

The test product was a proprietary mixture composed of baobab fruit pulp and acacia gum (200 mg/day), heat-killed whole cell *L. helveticus* HA-122 (with maltodextrin as carrier; 30 mg/d), and specific fractions of inactivated yeast strains *S. cerevisiae* AQP 12260, *S. cerevisiae* AQP 12988, and *C. jadinii* AQP 12549 (170 mg/d). The test product was provided by Virbac SA (Carros, France).

### SCIME model

**Figure 1.**
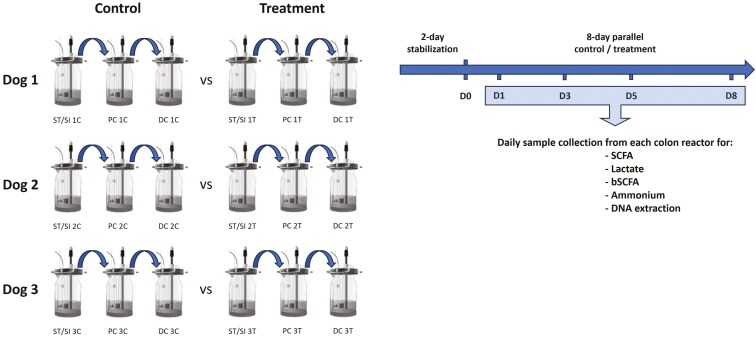
Schematic representation of the experimental design making use of a SCIME inoculated with the microbiota of 3 dogs with soft stools. The microbiota of each dog has been used to inoculate 2 systems, one acting as a control and the other treated with the test product. After 2 d of stabilization, the test product has been dosed in the ‘treatment’ arm of the study (D0). Samples for all analytical endpoints have been collected on D1, D3, D5, and D8 both in the proximal (PC) and distal colon (DC). bSCFA, branched short-chain fatty acid; D, day; DC, distal colon; PC, proximal colon; SCIME, Simulator of the Canine Intestinal Microbial Ecosystem; SCFA, short-chain fatty acid; ST/SI, stomach/small intestine reactor.

Given the high similarity between the human and canine intestinal conditions, the reactor setup for the SCIME model was based on the Simulator of the Human Intestinal Microbial Ecosystem (Molly et al., 1993). Details regarding the setup and validation of the SCIME model have been previously published (Duysburgh et al., 2020). The SCIME model used in the present study followed the setup of Duysburgh et al. with some modifications to account for the limited amount of time that a possibly dysbiotic microbial community linked to a gastrointestinal condition can be maintained in vitro. Briefly, the SCIME included 3 reactors representing the canine gastrointestinal tract (stomach and small intestine simulated in a single compartment and implemented by modifying conditions over time), the proximal colon (PC), and the distal colon (DC). The compartments were temperature controlled at 39 °C under anaerobic conditions, which was achieved by daily flushing with N_2_-gas. For each donor (*n* = 3), a control and treatment arm were executed in parallel rather than the sequential control and treatment periods used in the standard SCIME setup, in order to compare the evolution of the microbial communities in the presence and absence of a treatment (**Figure 1**). Colon reactors for the control and treatment arms were inoculated with appropriate fecal samples from each canine donor and the inoculum was allowed to grow and colonize the reactors for 2 d, while the reactors were fed with a basic SCIME nutritional medium (PDNM009, ProDigest, Belgium). After 2 d, the 8-d parallel control/treatment period was initiated. During this period, the reactors were fed with standard SCIME nutritional medium with or without test product supplementation to represent the treatment or control arms, respectively. Samples were collected from each colon vessel for analysis of microbial metabolic activity (lumen) and microbial community composition (lumen) on the first day (D1; after the first treatment was provided), third day (D3), fifth day (D5), and eighth day (D8) of the control/treatment period (**Figure 1**).

### Microbial metabolic activity

Quantitative analysis of the SCFAs, namely acetate, propionate, and butyrate, and of the bSCFAs (isobutyric acid, isovaleric acid, and isocaproic acid), was performed using capillary gas chromatography coupled with a flame ionization detector. SCFAs were isolated using liquid-liquid extraction with diethyl ether, after the addition of 2-methyl hexanoic acid as an internal standard (De Boever et al., 2000; De Weirdt et al., 2010). Lactate concentrations were measured using the Enzytec™ kit (R-Biopharm, Darmstadt, Germany) on an iCubio iMagic-M9 (Shenzhen iCubio Biomedical Technology Co. Ltd., Shenzhen, China), according to the manufacturer’s instructions. Ammonium analysis was run on an AQ300 Discrete Analyzer (Seal-Analytical, Rijen, The Netherlands) using the indophenol blue spectrophotometric method according to the manufacturer’s instructions. Each measurement was performed in a single repetition.

### Microbial community composition

Total DNA was isolated from the collected luminal samples as described by Duysburgh et al. (Duysburgh et al., 2019). Briefly: the DNA was extracted from a pellet of bacterial cells originating from a 1 mL sample after centrifugation for 5 min at 7,700 *g*. A Fastprep-24 device (MP BioMedicals, Illkirch, France) was used for homogenization, which was performed twice for 40 s at 4 m/s; the sample was allowed to rest for 5 min between shakings. 16S-targeted Illumina sequencing utilized primers that span 2 hypervariable regions (V3–V4) of the 16S rRNA gene profiling (341F, 5′-CCTACGGGNGGCWGCAG-3′; 785R, 5′-GACTACHVGGGTATCTAAKCC-3′; Klindworth et al., 2013). A pair-end sequencing approach was used to sequence 2 × 250 bp and 424 bp amplicons were generated (LGC Genomics GmbH, Berlin, Germany). Fragments of this size are considered taxonomically more informative than smaller fragments. The MiSeq SOP, as described by the Schloss lab, was followed for read assembly and cleanup (Schloss and Westcott, 2011; Kozich et al., 2013). Briefly, mothur (v.1.44.3) was used to assemble reads into contigs, perform alignment-based quality filtering (alignment to the mothur-reconstructed SILVA SEED alignment, v138), remove chimeras (vsearch v2.13.3), assign taxonomy using a naïve Bayesian classifier (Wang et al., 2007) and SILVA NR v138_1, and cluster contigs into operational taxonomic units at 97% sequence similarity. Sequences that could not be classified and those classified as Archaea, Eukaryota, mitochondria, or chloroplasts were removed. The representative sequence was the most abundant sequence within an OTU. Reads with maximum abundances ≤5 across samples were considered artifacts or bacteria with no biological impact and were thus removed.

Samples that were analyzed by 16S rRNA targeted gene sequencing were also analyzed using flow cytometry to determine the number of total bacterial cells. This allowed for the conversion of relative abundances into absolute abundances, which was done by multiplying the relative abundances of any population in a sample with the total cell count obtained for that sample using flow cytometry (Vandeputte et al., 2017). A BD Accuri C6 Plus Flow Cytometer (BD Biosciences, Franklin Lakes, New Jersey, USA) on the high flow rate was used to obtain cell counts. A threshold level of 700 on the SYTO channel was applied to separate bacterial cells from signal noise and medium debris. Parent and daughter gates were set as appropriate to determine all populations.

### Statistical analysis

For statistical comparisons of microbial community activity, 2o-way ANOVA for repeated measures were used to compare the effect of repeated administration between both groups (interaction). One-way ANOVA for repeated measures were used to assess the effect of repeated administration in each group followed by the Tukey HSD test in case of significance to check where the difference lay. Two-tailed paired *t*-tests were used for the comparison of the test product supplementation arm with the control arm within each donor. Differences were considered statistically significant with a *P*-value < 0.05.

Beta diversity analysis was conducted by hierarchically clustering Euclidean distances between samples using Ward’s minimum variance method. If absolute abundances were used (based on provided cell count data), a Variance Stabilizing Transformation was applied using DESeq2 v1.41.12 (Love et al., 2014) prior to the calculation of distances. Furthermore, a Discriminant Analysis of Principal Components (DAPC) plot was constructed with 2 discriminants and 80% of retained variance in the principal components using Adegenet v2.1.10 (Jombart, 2008; Jombart et al., 2010).

A hierarchical Differential Abundance Analysis was conducted to identify the taxa most likely to explain differences between study arms. This was run on the Trimmed Mean of M-values library sizes for each OTU using treeclimbR v0.1.5 and edgeR v3.42.4 (Huang et al., 2021). Benjamini–Hochberg multiple testing correction was used, and the alpha level was set at 0.05. Data from the treeclimbR analysis are shown in volcano plots, which are scatter plots that show statistical significance vs magnitude of change. The volcano plots classify the taxa into 4 distinct categories based on the abundance in compared study arms: not statistically significant and not biologically significant; biologically significant, but not statistically significant; statistically significant, but not biologically significant; or biologically and statistically significant. *P*-values ≤ 0.05 by the Kruskal–Wallis and Wilcoxon tests were considered statistically significant and a fold change (log2) > 2 was generally considered biologically relevant.

## Results

### Microbial metabolic activity

#### Measures of saccharolytic fermentation

**Figure 2.**
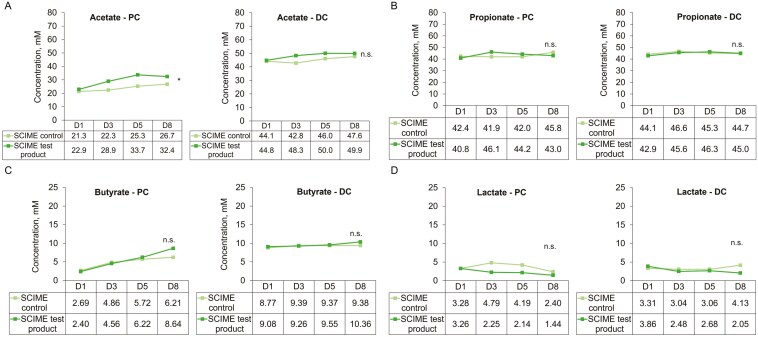
Microbial metabolic activity over time (D1, D3, D5, D8) in the proximal (PC) and distal colon (DC) of the SCIME is shown as changes in (A) acetate, (B) propionate, (C) butyrate, and (D) lactate concentrations. Each measurement was performed in a single repetition. Data for average values were derived using data from 3 canine donors. A 2-way ANOVA for repeated measures was used to compare the effect of repeated administration between control and treatment (interaction). *P < 0.05. D, day; DC, distal colon; PC, proximal colon; SCIME, Simulator of the Canine Intestinal Microbial Ecosystem.

In the PC, the acetate increased over time in both the test and control arms, with no difference between groups. The mean (SD) went from 21.3 (5.0) to 26.7 (4.1) mM in the control arm (+25%) and from 22.9 (8.0) to 29.1 (12.3) mM in the test arm (+27%), on days 1 and 8, respectively. Although the increase is lower with the control than with the supplement, it was significant only in the control group (*P* = 0.026, **Figure 2A**). There was however a significant difference between donors. In the DC, a similar increase over time was observed in both groups and tended to be significant with the test supplement (*P* = 0.073) but there was no difference between groups (**Figure 2A**). The mean (SD) went from 44.1 (4.6) to 47.6 (6.6) mM in the control arm (+8%) and from 44.8 (7.4) to 49.9 (4.3) mM in the test arm (+11%), on days 1 and 8, respectively. The difference between donors was also significant in the DC. SCFA and lactate levels for individual donors can be found in **Figure S1**. Acetate production was significantly increased with the test product compared with the control for donor B in the PC (*P* = 0.021), and DC (*P* = 0.020; **Figure S1A**). The production also tended to increase in donor C in the PC (*P* = 0.08).

Propionate production in the PC significantly differed in the 2 groups over time (*P* = 0.027). There was no significant change with the control (from 42.4 ± 3.3 to 45.8 ± 3.1 mM on D1 and D8, respectively) while the production tended to increase over time with the supplement (from 40.8 ± 0.5 to 43.0 ± 0.6 mM, respectively, *P* = 0.085), and especially on day 3 (46.1 ± 3.9 mM, + 13% vs day 1, *P* = 0.068) before decreasing again (**Figure 2B**). There was a significant difference between donors with the control but not with the test supplementation. In the DC, the production of propionate seemed to slightly increase in both arms on days 3 and 5, but there was no significant difference between time points and between groups. The mean (SD) went from 44.1 (3.4) to 46.6 (4.4) mM in the control arm (+ 6%) and from 42.8 (1.3) to 45.6 (4.1) mM in the test arm (+ 27%), on days 1 and 3, respectively. There was, however, a significant difference between donors in the control group. When considering the single donors, while propionate levels appeared numerically higher with the test product vs control for donor B (from D3), there was no significant difference in propionate levels between test product administration and the control for any donor in the PC (**Figure S1B**). In the DC, there was a significant decrease in propionate production with test product supplementation vs control for donor C (*P* = 0.044).

While butyrate levels seemed to increase over time in both groups in the PC and DC, there was no significant difference between time points and groups (**Figure 2C**). In the PC, the means (SD) went from 2.7 (4.2) to 6.2 (10.0) mM in the control arm (+ 131%) and from 2.4 (3.9) to 8.6 (9.2) mM in the test arm (+260%), on days 1 and 8, respectively. In the DC they went from 8.8 (7.7) to 9.4 (8.4) mM in the control arm (+7%) and from 9.1 (7.3) to 10.4 (7.7) mM in the test arm (+14%), respectively. There was however a significant difference between donors in both control and treated groups. Butyrate levels were not affected by the test product for donor A in either the PC or DC (**Figure S1C**). Donor B had low butyrate levels overall. Donor C had a strong butyrogenic response with test product supplementation at D8 in the PC while the analysis over the whole period did not show any statistical significance. Butyrate levels were unaffected by supplementation in the DC for this donor.

Finally, lactate levels seemed to decrease over time in both groups, especially in the treated group, but there was no difference between time points and groups in the PC and DC (**Figure 2D**). In the PC, the means (SD) went from 3.3 (2.9) to 2.4 (2.2) mM in the control arm (−27%) and from 3.3 (5.1) to 1.4 (2.0) mM in the test arm (−56%), on days 1 and 8, respectively. In the DC, they went from 3.3 (2.9) to 4.1 (6.6) mM in the control arm (+25%) and from 3.9 (4.2) to 2.0 (3.4) mM in the test arm (−47%), respectively. There was however a significant difference between donors in all groups, in the PC and DC. For donor A, lactate production was low throughout the study and regardless of test conditions (**Figure S1D**). Donors B and C seemed to have reduced lactate levels relative to control in both the PC and DC, but significant differences were only observed for donor C in the PC (*P* = 0.008).

#### Markers of proteolytic fermentation

**Figure 3.**
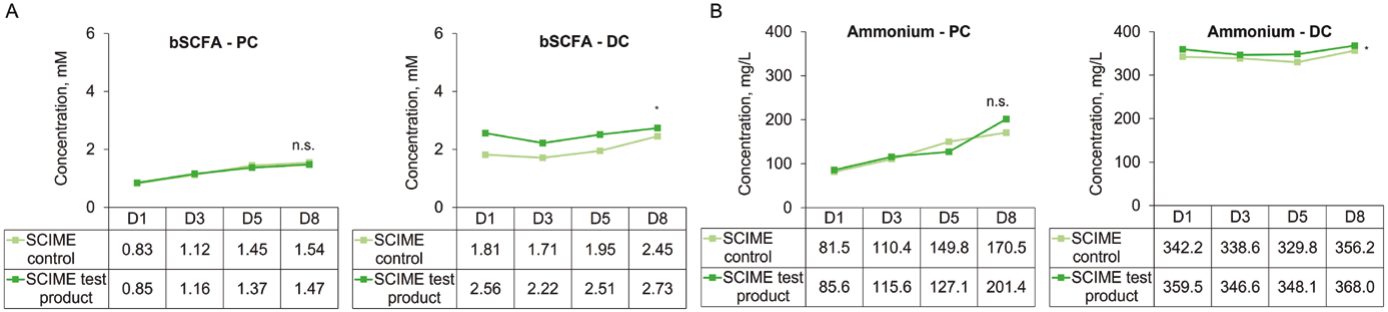
Microbial metabolic activity over time (D1, D3, D5, D8) in the proximal (PC) and distal colon (DC) of the SCIME is shown as changes in (A) bSCFA and (B) ammonium concentrations. Each measurement was performed in a single repetition. Data for average values were derived using data from 3 canine donors. A 2-way ANOVA for repeated measures were used to compare the effect of repeated administration between control and treatment (interaction). *P < 0.05. bSCFA, branched short-chain fatty acid; D, day; DC, distal colon; PC, proximal colon; SCIME, Simulator of the Canine Intestinal Microbial Ecosystem.

bSCFA levels seemed to increase in both groups but there was no difference between time points and groups, in the PC and DC (**Figure 3A**). Mean (SD) values went from 0.8 (1.3) to 1.5 (2.6) mM in the control arm (+87%) and from 0.8 (1.4) to 1.5 (2.4) mM in the test arm (+74%) in the PC, on days 1 and 8 respectively. In the DC, mean values went from 1.8 (2.1) to 2.5 (2.2) mM (+35%) and from 2.6 (1.9) to 2.7 (1.8) mM (+7%), respectively. There was again a significant difference between donors in both compartments. A significant increase in bSCFA was observed following product supplementation vs the control in the DC of donor B (*P* = 0.015; **Figure S2A**).

Ammonium levels significantly increased in both conditions in the PC, particularly on day 8 (*P* = 0.036 and 0.006 in the control and test conditions, respectively) with no difference between groups (**Figure 3B**). Mean (SD) values went from 81.3 (26.7) to 170.7 (88.6) mM in the control arm (+110%) and from 85.7 (17.6) to 201.7 (63.1) mM in the test arm (+135%) in the PC, on days 1 and 8, respectively. No difference between time points and conditions was observed in the DC. Mean (SD) values in the DC went from 342.0 (65.1) to 356.3 (69.2) mM in the control arm (+4%) and from 359.7 (1.9) to 368.0 (32.0) mM in the test arm (+2%); respectively. While donors A (in the PC and DC) and C (in the DC) seemed to have reduced ammonium levels with test product supplementation vs control, the level was significantly increased with test product supplementation in both the PC and DC of donor B (*P* = 0.019 and *P* = 0.001, respectively; **Figure S2B**).

### Microbial community composition

**Figure 4.**
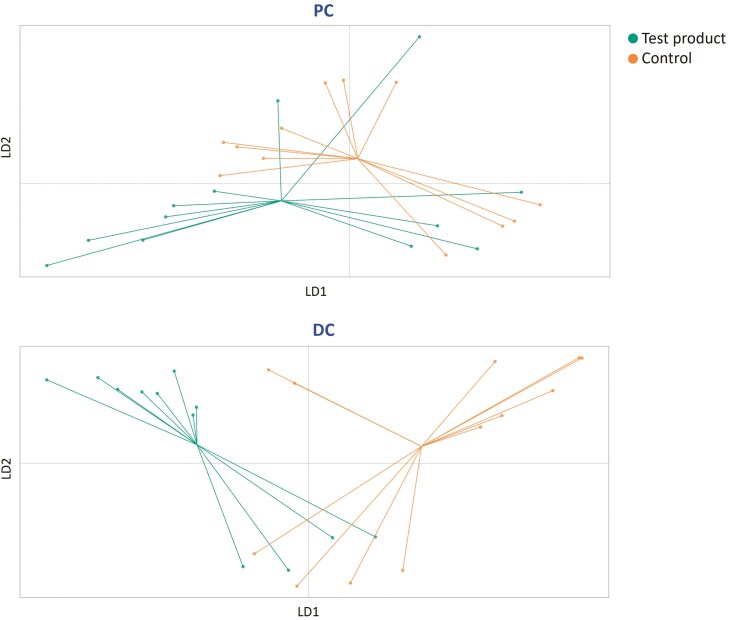
Discriminant analysis of principal components showing differences in community composition following treatment with the test product or control for the 3 canine donors in the SCIME model (days 1, 3, 5, and 8). Control, *n* = 12; test product, *n* = 12. Each dot represents one sample. DC, distal colon; PC, proximal colon; SCIME, Simulator of the Canine Intestinal Microbial Ecosystem

In the DAPC plots for the SCIME model, there was a clear shift with test product treatment vs control in both the PC and DC (**Figure 4**). Absolute levels of phyla and families for each single donor are shown in Tables S1, S2, and S3.

**Figure 5.**
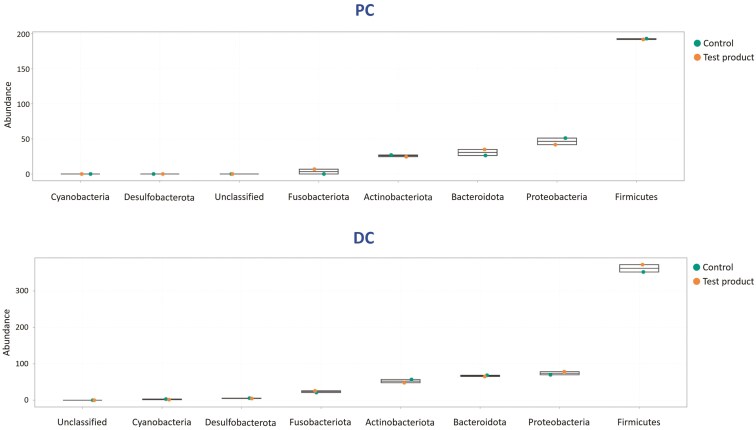
Jitter plots showing average abundances (log2 abundances) across all donors at the phylum level in the proximal (PC) and distal colon (DC). Data for average values were derived using data from 3 canine donors across all timepoints (D1, D3, D5, and D8). DC, distal colon; PC, proximal colon; SCIME, Simulator of the Canine Intestinal Microbial Ecosystem.

Firmicutes were the most abundant phylum in both colon regions (**Figure 5**). During test product supplementation vs the control, the abundance of Bacteroidota and Fusobacteriota increased, while that of Proteobacteria decreased in the PC, and the abundance of Firmicutes increased in the DC.

**Figure 6.**
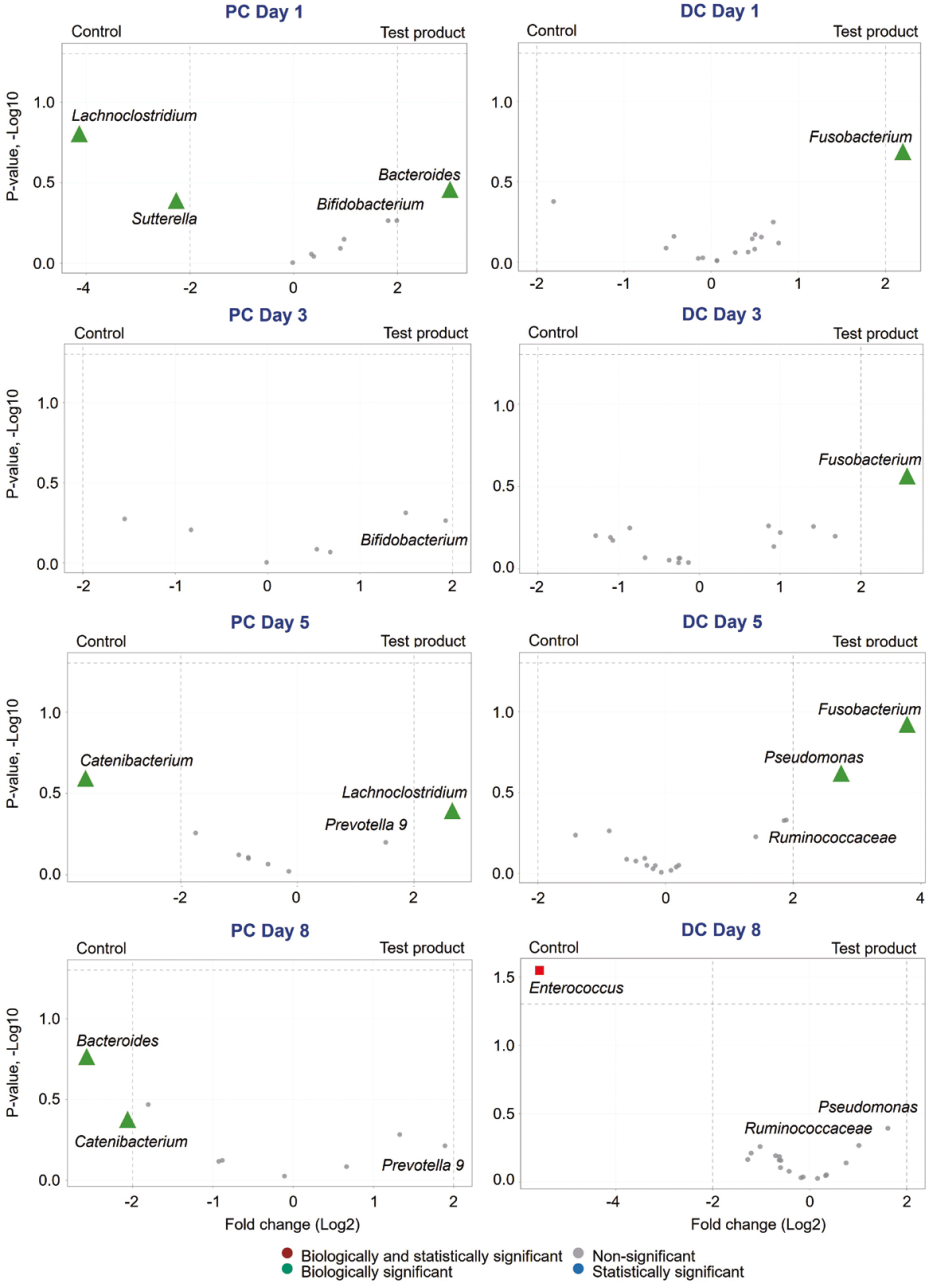
Differential abundance analysis at days 1, 3, 5, and 8 in the proximal (PC) and distal colon (DC) across 3 canine donors using treeclimbR analysis. The analysis is based on relative abundance data (total sum scaling). The scatter plot classifies taxa into 4 categories based on abundances in the compared conditions: biologically and statistically significant (square), biologically significant but not statistically significant (triangle), neither biologically nor statistically significant (round), or statistically significant but not biologically significant (star) for the difference between test product and control. DC, distal colon; PC, proximal colon; SCIME, Simulator of the Canine Intestinal Microbial Ecosystem.

treeclimbR analysis (i.e., differential abundance analysis) was used to identify which bacterial groups were responsible for the significant and/or biologically relevant changes observed in microbial community composition. Across the different donors tested, bacterial groups involved in primary substrate degradation were enriched in the PC following test product supplementation compared with the control. *Bifidobacterium* was enriched at D1 and D3, reaching levels close to biological relevance. *Prevotella* abundance was enriched with test product supplementation towards the end of the treatment period (reaching levels close to biological significance) mainly at the expense of the biologically reduced *Bacteroides*. Furthermore, *Sutterella*, a member of the Proteobacteria phylum, had a biologically significant lower abundance at D1 following product supplementation (**Figure 6**). In the DC, the butyrate-producing genus *Fusobacterium* was consistently enriched, reaching biological relevance at D1, D3, and D5, and the butyrate-producing *Ruminococcaceae* family was enriched, reaching levels close to biological relevance at D5 and D8, with test product supplementation vs control (**Figure 6**).

## Discussion

Overall, this study suggested an increase in microbial fermentation in the gut microbiome of canine donors with soft stools following supplementation with the test product. Propionate levels increased in the PC early after supplementation. Acetate and butyrate were also increased, although more in a donor-dependent manner while lactate levels tended to be decreased with supplementation compared to control. The health-promoting *Bifidobacterium* and *Prevotella* genera were mainly responsible for the observed increase in microbial fermentation.

Lactate and SCFAs, including acetate, propionate, and butyrate are byproducts of saccharolytic fermentation in the colon; their levels reflect overall fermentation by the gut microbiota. Lactate can be converted to butyrate and/or propionate by lactogenic microbes (Culp and Goodman, 2023). Lower levels of lactate following treatment can be considered as an indicator of efficient cross-feeding interactions within the microbial community. Supplementation with the test product resulted in increased propionate and acetate production, although not to a significant level for the latter. The increase in both SCFAs was mainly observed in the PC indicating that the test product can be easily fermented. Butyrate levels appeared to increase later in the supplementation period, particularly in the PC, but the changes were not significant upon considering the entire treatment period. Butyrate is an important product of microbial fermentation, as it is a primary source of energy for epithelial cells and helps to maintain a healthy intestinal barrier (Martin-Gallausiaux et al., 2021).

The effect on SCFA production varied a lot, depending on the gut microbiota of the selected donors. Individual donor results indicated that there may be potential for test product supplementation to have a propiogenic and butyrogenic effect in some specific canine donors. It has been shown that dogs with serious GI problems (i.e., inflammatory bowel disease) have significantly lower levels of fecal acetate, propionate, and butyrate compared with healthy dogs (Minamoto et al., 2019). Thus, the test product-dependent increase in propionate and butyrate production, for some individual donors, can be considered a beneficial effect that must be further investigated with a higher number of donors.

bSCFAs and ammonium are indicators of proteolytic fermentation, which is generally considered less desirable because the process involves the production of highly toxic compounds (Diether and Willing, 2019). In the PC for the SCIME model, changes in bSCFA and ammonium levels were similar to the test product and control. Significant increases vs control were observed for both parameters (in the DC mainly) in only one donor (B) and the levels of both bSCFA and ammonium remained within the normal physiological range (Duysburgh et al., 2020; Van den Abbeele et al., 2020).

DAPC plots evaluating beta diversity showed distinct clustering of the test product and control samples, indicating changes in the microbial community composition with repeated administration of the test product. Indeed, microbial community composition analysis demonstrated that primary substrate degraders involved in saccharolytic fermentation were enriched following repeated administration of the test product and provided insight into the mechanisms behind the increases in specific SCFAs. The increased abundance of the Bacteroidota phylum with test product supplementation vs control likely explains the increased propionate and acetate production (donor-dependent), as both SCFAs are mainly produced by bacteria in this phylum (Garcia-Lopez et al., 2019). The treatment with the test product also increased the concentration of Firmicutes and of Fusobacteriota phylum (the latter especially for Donor C). Most butyrate-producing bacteria are members of the Firmicutes phylum (Singh et al., 2022) and members of the Fusobacteriota phylum are also butyrate-producers. A positive effect on butyrate production was only observed with donor C, thus again highlighting the importance of interindividual variability in assessing the modulation of the gut microbiota. In dogs, Fusobacteriota are severely impacted by gastrointestinal diseases and their recovery is slower than other phyla (Pilla and Suchodolski, 2019; Pilla et al., 2020). Attempting to increase Fusobacteriota using specific food ingredients, such as those found in the test product, maybe a useful strategy for improving gastrointestinal diseases. Furthermore, the strong enrichment of *Bifidobacterium* early in the SCIME study reached levels close to biological relevance. *Bifidobacterium* are considered beneficial saccharolytic bacteria with versatile fiber-degrading potential. They mainly produce acetate and lactate, which are important drivers of cross-feeding interactions with other bacteria that result in the production of health-promoting metabolites such as propionate and butyrate (Riviere et al., 2016). Thus, the enrichment of *Bifidobacterium* is associated with a potential health benefit.

The Proteobacteria phylum is mostly linked with enteric diseases as it contains several opportunistic pathogenic microorganisms, as well as commensal bacteria involved in proteolytic fermentation (Rizzatti et al., 2017). The reduced abundance of Proteobacteria observed with test product administration strengthens the conclusion that primary substrate degraders involved in saccharolytic fermentation were enriched following repeated administration of the test product, thereby reducing proteolytic fermentation as indicated by the reduction in ammonium production in some donors.

As with any in vitro study, the study findings are limited in that they cannot be linked directly to in vivo effects. Future studies of the test product in dogs with a defined dysbiosis should therefore be conducted to confirm these in vitro findings. Considering the individual donor data, the study findings indicate that there were some donor-dependent differences in the test product effects. This suggests that the effects may be dependent on the level of specific microbiome composition of an individual dog and as such may not be generalizable to all dogs.

In conclusion, using in vitro models inoculated with the fecal microbiota of dogs with soft stools, it was demonstrated that test product supplementation improved SCFA production, increased gut microbiome diversity, and enhanced the growth of several gut bacteria associated with health benefits to the host. Changes in SCFA production could be linked with changes in specific microbial abundance. The changes observed in this pilot study are indicative of the fact that repeated administrations of the test product may have the potential to reduce dysbiosis-associated symptoms in dogs with moderate digestive problems, such as soft stools, and that this potential must be further investigated in terms of interindividual variability following repeated administration.

## Supplementary Material

skaf056_suppl_Supplementary_Materials

## Abbreviations

bSCFA: branched short-chain fatty acid
DAPC: discriminant analysis of principal components
DC: distal colon
M-SCIME: Mucosal Simulator of the Canine Intestinal Microbial Ecosystem
OTU: operational taxonomic Units
PC: proximal colon
SCFA: short-chain fatty acid
SCIME: Simulator of the Canine Intestinal Microbial Ecosystem

## References

[CIT0001] Arpaia, N., C. Campbell, X. Fan, S. Dikiy, J. van der Veeken, P. deRoos, H. Liu, J. R. Cross, K. Pfeffer, P. J. Coffer, et al 2013. Metabolites produced by commensal bacteria promote peripheral regulatory T-cell generation. Nature 504:451–455. doi: https://doi.org/10.1038/nature12726 24226773 PMC3869884

[CIT0002] Calame, W., A. R. Weseler, C. Viebke, C. Flynn, and A. D. Siemensma. 2008. Gum arabic establishes prebiotic functionality in healthy human volunteers in a dose-dependent manner. Br. J. Nutr. 100:1269–1275. doi: https://doi.org/10.1017/S0007114508981447 18466655

[CIT0003] Culp, E. J., and A. L. Goodman. 2023. Cross-feeding in the gut microbiome: Ecology and mechanisms. Cell Host Microbe 31:485–499. doi: https://doi.org/10.1016/j.chom.2023.03.016 37054671 PMC10125260

[CIT0004] De Boever, P., B. Deplancke, and W. Verstraete. 2000. Fermentation by gut microbiota cultured in a simulator of the human intestinal microbial ecosystem is improved by supplementing a soygerm powder. J. Nutr. 130:2599–2606. doi: https://doi.org/10.1093/jn/130.10.2599 11015496

[CIT0006] Deleu, S., K. Machiels, J. Raes, K. Verbeke, and S. Vermeire. 2021. Short chain fatty acids and its producing organisms: an overlooked therapy for IBD? EBioMedicine 66:103293. doi: https://doi.org/10.1016/j.ebiom.2021.103293 33813134 PMC8047503

[CIT0005] De Weirdt, R., S. Possemiers, G. Vermeulen, T. C. Moerdijk-Poortvliet, H. T. Boschker, W. Verstraete, and T. Van de Wiele. 2010. Human faecal microbiota display variable patterns of glycerol metabolism. FEMS Microbiol. Ecol. 74:601–611. doi: https://doi.org/10.1111/j.1574-6941.2010.00974.x 20946352

[CIT0007] Diether, N. E., and B. P. Willing. 2019. Microbial fermentation of dietary protein: an important factor in diet(-)microbe(-)host interaction. Microorganisms. 7:19. doi: https://doi.org/10.3390/microorganisms7010019 30642098 PMC6352118

[CIT0009] Duysburgh, C., P. V. Abbeele, K. Krishnan, T. F. Bayne, and M. Marzorati. 2019. A synbiotic concept containing spore-forming Bacillus strains and a prebiotic fiber blend consistently enhanced metabolic activity by modulation of the gut microbiome in vitro. Int. J. Pharm. X. 1:100021. doi: https://doi.org/10.1016/j.ijpx.2019.100021 31517286 PMC6733369

[CIT0008] Duysburgh, C., W. P. Ossieur, K. De Paepe, P. V. Abbeele, R. Vichez-Vargas, M. Vital, D. H. Pieper, T. Van de Wiele, M. Hesta, S. Possemiers, et al 2020. Development and validation of the Simulator of the Canine Intestinal Microbial Ecosystem (SCIME)1. J. Anim. Sci. 98:skz357. doi: https://doi.org/10.1093/jas/skz357 31768533 PMC6986438

[CIT0010] Foltz, M., A. C. Zahradnik, P. V. Abbeele, J. Ghyselinck, and M. Marzorati. 2021. A pectin-rich, baobab fruit pulp powder exerts prebiotic potential on the human gut microbiome in vitro. Microorganisms. 9:1981. doi: https://doi.org/10.3390/microorganisms9091981 34576876 PMC8467054

[CIT0011] Garcia-Lopez, M., J. P. Meier-Kolthoff, B. J. Tindall, S. Gronow, T. Woyke, N. C. Kyrpides, R. L. Hahnke, and M. Goker. 2019. Analysis of 1,000 type-strain genomes improves taxonomic classification of Bacteroidetes. Front. Microbiol. 10:2083. doi: https://doi.org/10.3389/fmicb.2019.02083 31608019 PMC6767994

[CIT0012] Gozdzik, P., F. Magkos, T. Sledzinski, and A. Mika. 2023. Monomethyl branched-chain fatty acids: health effects and biological mechanisms. Prog. Lipid. Res. 90:101226. doi: https://doi.org/10.1016/j.plipres.2023.101226 37094753

[CIT0013] Guan, X., F. Molist, F. Bravo de Laguna, and D. Saornil. 2017. Effect of the combination of three yeast strains on post weaning piglets after an experimental E. coli infection. Anim. Produc. Sci. 57:2498–2498. doi: https://doi.org/10.1071/anv57n12ab010

[CIT0014] Hand, D., C. Wallis, A. Colyer, and C. W. Penn. 2013. Pyrosequencing the canine faecal microbiota: breadth and depth of biodiversity. PLoS One 8:e53115. doi: https://doi.org/10.1371/journal.pone.0053115 23382835 PMC3561364

[CIT0015] Hoefman, S., A. Pommerening-Roser, E. Samyn, P. De Vos, and K. Heylen. 2013. Efficient cryopreservation protocol enables accessibility of a broad range of ammonia-oxidizing bacteria for the scientific community. Res. Microbiol. 164:288–292. doi: https://doi.org/10.1016/j.resmic.2013.01.007 23376087

[CIT0016] Honneffer, J. B., J. M. Steiner, J. A. Lidbury, and J. S. Suchodolski. 2017. Variation of the microbiota and metabolome along the canine gastrointestinal tract. Metabolomics. 13:26. doi: https://doi.org/10.1007/s11306-017-1165-3.

[CIT0017] Huang, R., C. Soneson, P. L. Germain, T. S. B. Schmidt, C. V. Mering, and M. D. Robinson. 2021. treeclimbR pinpoints the data-dependent resolution of hierarchical hypotheses. Genome Biol. 22:157. doi: https://doi.org/10.1186/s13059-021-02368-1 34001188 PMC8127214

[CIT0018] Jombart, T. 2008. adegenet: a R package for the multivariate analysis of genetic markers. Bioinformatics 24:1403–1405. doi: https://doi.org/10.1093/bioinformatics/btn129 18397895

[CIT0019] Jombart, T., S. Devillard, and F. Balloux. 2010. Discriminant analysis of principal components: a new method for the analysis of genetically structured populations. BMC Genet. 11:94. doi: https://doi.org/10.1186/1471-2156-11-94 20950446 PMC2973851

[CIT0020] Klindworth, A., E. Pruesse, T. Schweer, J. Peplies, C. Quast, M. Horn, and F. O. Glockner. 2013. Evaluation of general 16S ribosomal RNA gene PCR primers for classical and next-generation sequencing-based diversity studies. Nucleic Acids Res. 41:e1. doi: https://doi.org/10.1093/nar/gks808 22933715 PMC3592464

[CIT0021] Kozich, J. J., S. L. Westcott, N. T. Baxter, S. K. Highlander, and P. D. Schloss. 2013. Development of a dual-index sequencing strategy and curation pipeline for analyzing amplicon sequence data on the MiSeq Illumina sequencing platform. Appl. Environ. Microbiol. 79:5112–5120. doi: https://doi.org/10.1128/AEM.01043-13 23793624 PMC3753973

[CIT0022] Lee, J. J., H. Kyoung, J. H. Cho, J. Choe, Y. Kim, Y. Liu, J. Kang, H. Lee, H. B. Kim, and M. Song. 2021. Dietary yeast cell wall improves growth performance and prevents of diarrhea of weaned pigs by enhancing gut health and anti-inflammatory immune responses. Animals (Basel). 11:2269. doi: https://doi.org/10.3390/ani11082269 34438727 PMC8388398

[CIT0023] Love, M. I., W. Huber, and S. Anders. 2014. Moderated estimation of fold change and dispersion for RNA-seq data with DESeq2. Genome Biol. 15:550. doi: https://doi.org/10.1186/s13059-014-0550-8 25516281 PMC4302049

[CIT0024] Makki, K., E. C. Deehan, J. Walter, and F. Backhed. 2018. The impact of dietary fiber on gut microbiota in host health and disease. Cell Host Microbe 23:705–715. doi: https://doi.org/10.1016/j.chom.2018.05.012 29902436

[CIT0026] Martinelli, M., D. Ummarino, F. P. Giugliano, E. Sciorio, C. Tortora, D. Bruzzese, D. De Giovanni, I. Rutigliano, S. Valenti, C. Romano, et al 2017. Efficacy of a standardized extract of Matricariae chamomilla L., Melissa officinalis L. and tyndallized Lactobacillus acidophilus (HA122) in infantile colic: an open randomized controlled trial. Neurogastroenterol. Motil. 29:e13145. doi: https://doi.org/10.1111/nmo.13145 28665038

[CIT0025] Martin-Gallausiaux, C., L. Marinelli, H. M. Blottiere, P. Larraufie, and N. Lapaque. 2021. SCFA: mechanisms and functional importance in the gut. Proc. Nutr. Soc. 80:37–49. doi: https://doi.org/10.1017/S0029665120006916 32238208

[CIT0027] Middelbos, I. S., B. M. Vester Boler, A. Qu, B. A. White, K. S. Swanson, and G. C. Fahey, Jr. 2010. Phylogenetic characterization of fecal microbial communities of dogs fed diets with or without supplemental dietary fiber using 454 pyrosequencing. PLoS One 5:e9768. doi: https://doi.org/10.1371/journal.pone.0009768 20339542 PMC2842427

[CIT0028] Minamoto, Y., T. Minamoto, A. Isaiah, P. Sattasathuchana, A. Buono, V. R. Rangachari, I. H. McNeely, J. Lidbury, J. M. Steiner, and J. S. Suchodolski. 2019. Fecal short-chain fatty acid concentrations and dysbiosis in dogs with chronic enteropathy. J. Vet. Intern. Med. 33:1608–1618. doi: https://doi.org/10.1111/jvim.15520 31099928 PMC6639498

[CIT0029] Molly, K., M. Vande Woestyne, and W. Verstraete. 1993. Development of a 5-step multi-chamber reactor as a simulation of the human intestinal microbial ecosystem. Appl. Microbiol. Biotechnol. 39:254–258. doi: https://doi.org/10.1007/BF00228615 7763732

[CIT0030] Morrison, D. J., and T. Preston. 2016. Formation of short chain fatty acids by the gut microbiota and their impact on human metabolism. Gut. Microbes. 7:189–200. doi: https://doi.org/10.1080/19490976.2015.1134082 26963409 PMC4939913

[CIT0032] Pilla, R., and J. S. Suchodolski. 2019. The role of the canine gut microbiome and metabolome in health and gastrointestinal disease. Front. Vet. Sci. 6:498. doi: https://doi.org/10.3389/fvets.2019.00498 31993446 PMC6971114

[CIT0033] Pilla, R., and J. S. Suchodolski. 2021. The gut microbiome of dogs and cats, and the influence of diet. Vet. Clin. North Am. Small Anim. Pract. 51:605–621. doi: https://doi.org/10.1016/j.cvsm.2021.01.002 33653538

[CIT0031] Pilla, R., F. P. Gaschen, J. W. Barr, E. Olson, J. Honneffer, B. C. Guard, A. B. Blake, D. Villanueva, M. R. Khattab, M. K. AlShawaqfeh, et al 2020. Effects of metronidazole on the fecal microbiome and metabolome in healthy dogs. J. Vet. Intern. Med. 34:1853–1866. doi: https://doi.org/10.1111/jvim.15871 32856349 PMC7517498

[CIT0034] Pique, N., M. Berlanga, and D. Minana-Galbis. 2019. Health benefits of heat-killed (tyndallized) probiotics: an overview. Int. J. Mol. Sci. 20:2534. doi: https://doi.org/10.3390/ijms20102534 31126033 PMC6566317

[CIT0035] Priyadarshini, M., K. U. Kotlo, P. K. Dudeja, and B. T. Layden. 2018. Role of short chain fatty acid receptors in intestinal physiology and pathophysiology. Compr. Physiol. 8:1091–1115. doi: https://doi.org/10.1002/cphy.c170050 29978895 PMC6058973

[CIT0038] Rawling, M. D., N. Pontefract, A. Rodiles, I. Anagnostara, E. Leclercq, M. Schiavone, M. Castex, and D. L. Merrifield. 2019. The effect of feeding a novel multistrain yeast fraction on European seabass (Dicentrachus labrax) intestinal health and growth performance. J. World Aquacult. Soc. 50:1108–1122. doi: https://doi.org/10.1111/jwas.12591

[CIT0036] Rawling, M., E. Leclercq, A. Foey, M. Castex, and D. Merrifield. 2021. A novel dietary multi-strain yeast fraction modulates intestinal toll-like-receptor signalling and mucosal responses of rainbow trout (Oncorhynchus mykiss). PLoS One 16:e0245021. doi: https://doi.org/10.1371/journal.pone.0245021 33434201 PMC7802930

[CIT0037] Rawling, M., M. Schiavone, A. Mugnier, E. Leclercq, D. Merrifield, A. Foey, and E. Apper. 2023. Modulation of zebrafish (Danio rerio) intestinal mucosal barrier function fed different postbiotics and a probiotic from Lactobacilli. Microorganisms. 11:2900. doi: https://doi.org/10.3390/microorganisms11122900 38138044 PMC10745996

[CIT0039] Riviere, A., M. Selak, D. Lantin, F. Leroy, and L. De Vuyst. 2016. Bifidobacteria and butyrate-producing colon bacteria: importance and strategies for their stimulation in the human gut. Front. Microbiol. 7:979. doi: https://doi.org/10.3389/fmicb.2016.00979 27446020 PMC4923077

[CIT0040] Rizzatti, G., L. R. Lopetuso, G. Gibiino, C. Binda, and A. Gasbarrini. 2017. Proteobacteria: a common factor in human diseases. Biomed Res. Int. 2017:9351507. doi: https://doi.org/10.1155/2017/9351507 29230419 PMC5688358

[CIT0041] Salminen, S., M. C. Collado, A. Endo, C. Hill, S. Lebeer, E. M. M. Quigley, M. E. Sanders, R. Shamir, J. R. Swann, H. Szajewska, et al 2021. The International Scientific Association of Probiotics and Prebiotics (ISAPP) consensus statement on the definition and scope of postbiotics. Nat. Rev. Gastroenterol. Hepatol. 18:649–667. doi: https://doi.org/10.1038/s41575-021-00440-6 33948025 PMC8387231

[CIT0042] Schiavone, M., A. Vax, C. Formosa, H. Martin-Yken, E. Dague, and J. M. Francois. 2014. A combined chemical and enzymatic method to determine quantitatively the polysaccharide components in the cell wall of yeasts. FEMS Yeast Res. 14:933–947. doi: https://doi.org/10.1111/1567-1364.12182 25041403

[CIT0043] Schloss, P. D., and S. L. Westcott. 2011. Assessing and improving methods used in operational taxonomic unit-based approaches for 16S rRNA gene sequence analysis. Appl. Environ. Microbiol. 77:3219–3226. doi: https://doi.org/10.1128/AEM.02810-10 21421784 PMC3126452

[CIT0044] Singh, V., G. Lee, H. Son, H. Koh, E. S. Kim, T. Unno, and J. H. Shin. 2022. Butyrate producers, “The Sentinel of Gut”: Their intestinal significance with and beyond butyrate, and prospective use as microbial therapeutics. Front. Microbiol. 13:1103836. doi: https://doi.org/10.3389/fmicb.2022.1103836 36713166 PMC9877435

[CIT0045] Suchodolski, J. S. 2011. Companion animals symposium: microbes and gastrointestinal health of dogs and cats. J. Anim. Sci. 89:1520–1530. doi: https://doi.org/10.2527/jas.2010-3377 21075970 PMC7199667

[CIT0046] Suchodolski, J. S., J. Camacho, and J. M. Steiner. 2008. Analysis of bacterial diversity in the canine duodenum, jejunum, ileum, and colon by comparative 16S rRNA gene analysis. FEMS Microbiol. Ecol. 66:567–578. doi: https://doi.org/10.1111/j.1574-6941.2008.00521.x 18557939

[CIT0047] Suchodolski, J. S., M. E. Markel, J. F. Garcia-Mazcorro, S. Unterer, R. M. Heilmann, S. E. Dowd, P. Kachroo, I. Ivanov, Y. Minamoto, E. M. Dillman, et al 2012. The fecal microbiome in dogs with acute diarrhea and idiopathic inflammatory bowel disease. PLoS One 7:e51907. doi: https://doi.org/10.1371/journal.pone.0051907 23300577 PMC3530590

[CIT0048] Sunvold, G. D., H. S. Hussein, G. C. Fahey, Jr, N. R. Merchen, and G. A. Reinhart. 1995. In vitro fermentation of cellulose, beet pulp, citrus pulp, and citrus pectin using fecal inoculum from cats, dogs, horses, humans, and pigs and ruminal fluid from cattle. J. Anim. Sci. 73:3639–3648. doi: https://doi.org/10.2527/1995.73123639x 8655439

[CIT0049] Taverniti, V., and S. Guglielmetti. 2012. Health-promoting properties of Lactobacillus helveticus. Front. Microbiol. 3:392. doi: https://doi.org/10.3389/fmicb.2012.00392 23181058 PMC3500876

[CIT0050] Thorakkattu, P., A. C. Khanashyam, K. Shah, K. S. Babu, A. S. Mundanat, A. Deliephan, G. S. Deokar, C. Santivarangkna, and N. P. Nirmal. 2022. Postbiotics: current trends in food and pharmaceutical industry. Foods. 11:3094. doi: https://doi.org/10.3390/foods11193094 36230169 PMC9564201

[CIT0051] Van den Abbeele, P., C. Duysburgh, M. Rakebrandt, and M. Marzorati. 2020. Dried yeast cell walls high in beta-glucan and mannan-oligosaccharides positively affect microbial composition and activity in the canine gastrointestinal tract in vitro. J. Anim. Sci. 98:skaa173. doi: https://doi.org/10.1093/jas/skaa173 32497185 PMC7295327

[CIT0052] Vandeputte, D., G. Kathagen, K. D’Hoe, S. Vieira-Silva, M. Valles-Colomer, J. Sabino, J. Wang, R. Y. Tito, L. De Commer, Y. Darzi, et al 2017. Quantitative microbiome profiling links gut community variation to microbial load. Nature 551:507–511. doi: https://doi.org/10.1038/nature24460 29143816

[CIT0053] Wang, Q., G. M. Garrity, J. M. Tiedje, and J. R. Cole. 2007. Naive Bayesian classifier for rapid assignment of rRNA sequences into the new bacterial taxonomy. Appl. Environ. Microbiol. 73:5261–5267. doi: https://doi.org/10.1128/AEM.00062-07 17586664 PMC1950982

[CIT0054] Wang, S. P., L. A. Rubio, S. H. Duncan, G. E. Donachie, G. Holtrop, G. Lo, F. M. Farquharson, J. Wagner, J. Parkhill, P. Louis, et al 2020. Pivotal roles for pH, lactate, and lactate-utilizing bacteria in the stability of a human colonic microbial ecosystem. mSystems 5:e00645–e00620. doi: https://doi.org/10.1128/mSystems.00645-20 PMC7483512 32900872

[CIT0055] Xue, G. D., S. B. Wu, M. Choct, and R. A. Swick. 2017. Effects of yeast cell wall on growth performance, immune responses and intestinal short chain fatty acid concentrations of broilers in an experimental necrotic enteritis model. Anim. Nutr. 3:399–405. doi: https://doi.org/10.1016/j.aninu.2017.08.002 29767160 PMC5941278

