# Supplementary material for: A specific blend of prebiotics and postbiotics improved the gut microbiome of dogs with soft stools in the in vitro Simulator of the Canine Intestinal Microbial Ecosystem
Source: J Anim Sci. 2025 Feb 27;103:skaf056. doi: 10.1093/jas/skaf056 (PMC11971633; doi:10.1093/jas/skaf056)
Supplement: skaf056_suppl_Supplementary_Materials [file skaf056_suppl_supplementary_materials.pdf]

Supplemental Material

A specific blend of prebiotics and postbiotics improved gut microbiome dysbiosis in the *in vitro* Simulator of the Canine Intestinal Microbial Ecosystem (SCIME)

Cindy Duysburgh, *et al*.

Figure S1. Microbial metabolic activity over time (D1, D3, D5, D8) in the proximal (PC) and distal colon (DC) for the SCIME study shown as changes in (A) acetate, (B) propionate, (C) butyrate, and (D) lactate concentrations for individual canine donors (Donors A-C). Each measurement was performed in single repetition. Two-tailed paired t-tests were used to determine significant differences between test product and control. *P <0.05.
D, day; DC, distal colon; n.s., not significant; PC, proximal colon; SCIME, Simulator of the Canine Intestinal Microbial Ecosystem

A

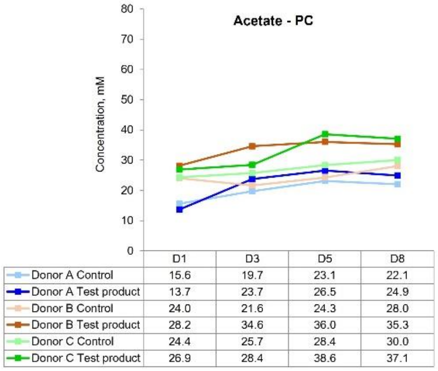

	D1	D3	D5	D8	
Donor A Control	15.6	19.7	23.1	22.1	n.s.
Donor A Test product	13.7	23.7	26.5	24.9	
Donor B Control	24.0	21.6	24.3	28.0	*
Donor B Test product	28.2	34.6	36.0	35.3	
Donor C Control	24.4	25.7	28.4	30.0	n.s.
Donor C Test product	26.9	28.4	38.6	37.1	

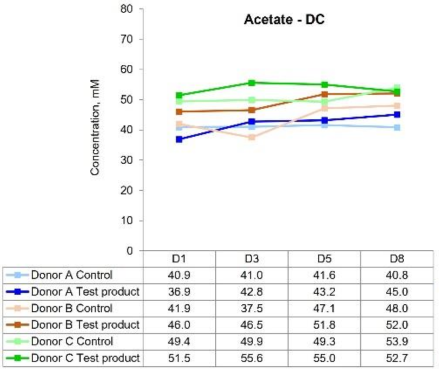

	D1	D3	D5	D8	
Donor A Control	40.9	41.0	41.6	40.8	n.s.
Donor A Test product	36.9	42.8	43.2	45.0	
Donor B Control	41.9	37.5	47.1	48.0	*
Donor B Test product	46.0	46.5	51.8	52.0	
Donor C Control	49.4	49.9	49.3	53.9	n.s.
Donor C Test product	51.5	55.6	55.0	52.7	

B

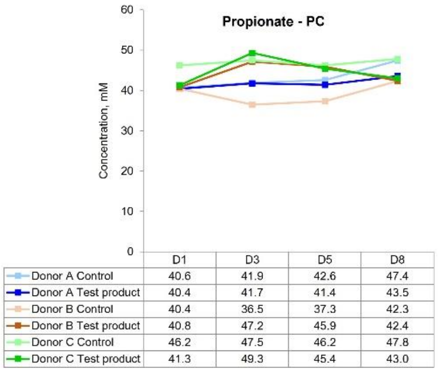

	D1	D3	D5	D8	
Donor A Control	40.6	41.9	42.6	47.4	n.s.
Donor A Test product	40.4	41.7	41.4	43.5	
Donor B Control	40.4	36.5	37.3	42.3	n.s.
Donor B Test product	40.8	47.2	45.9	42.4	
Donor C Control	46.2	47.5	46.2	47.8	n.s.
Donor C Test product	41.3	49.3	45.4	43.0	

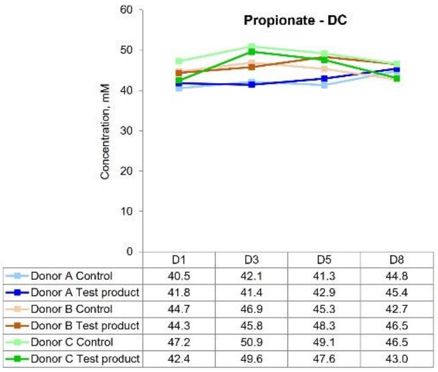

	D1	D3	D5	D8	
Donor A Control	40.5	42.1	41.3	44.8	n.s.
Donor A Test product	41.8	41.4	42.9	45.4	
Donor B Control	44.7	46.9	45.3	42.7	n.s.
Donor B Test product	44.3	45.8	48.3	46.5	
Donor C Control	47.2	50.9	49.1	46.5	*
Donor C Test product	42.4	49.6	47.6	43.0	

C

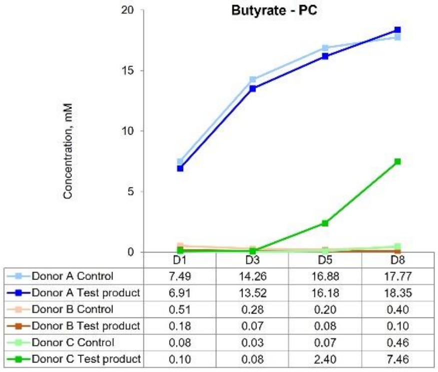

	D1	D3	D5	D8	
Donor A Control	7.49	14.26	16.88	17.77	n.s.
Donor A Test product	6.91	13.52	16.18	18.35	
Donor B Control	0.51	0.28	0.20	0.40	*
Donor B Test product	0.18	0.07	0.08	0.10	
Donor C Control	0.08	0.03	0.07	0.46	n.s.
Donor C Test product	0.10	0.08	2.40	7.46	

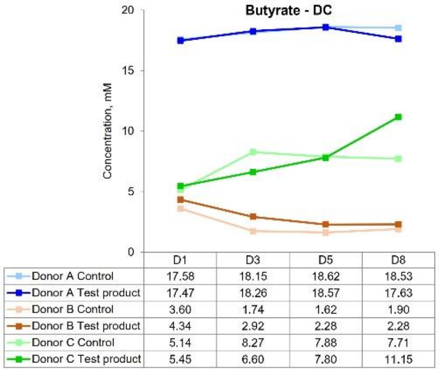

	D1	D3	D5	D8	
Donor A Control	17.58	18.15	18.62	18.53	n.s.
Donor A Test product	17.47	18.26	18.57	17.63	
Donor B Control	3.60	1.74	1.62	1.90	*
Donor B Test product	4.34	2.92	2.28	2.28	
Donor C Control	5.14	8.27	7.88	7.71	n.s.
Donor C Test product	5.45	6.60	7.80	11.15	

D

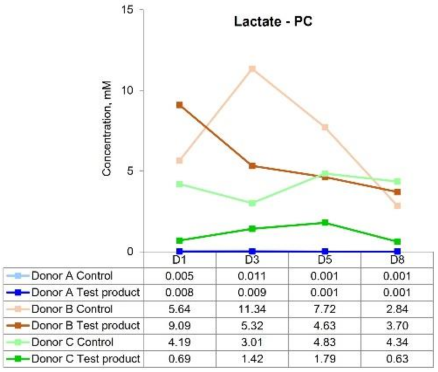

	D1	D3	D5	D8	
Donor A Control	0.005	0.011	0.001	0.001	n.s.
Donor A Test product	0.008	0.009	0.001	0.001	
Donor B Control	5.64	11.34	7.72	2.84	n.s.
Donor B Test product	9.09	5.32	4.63	3.70	
Donor C Control	4.19	3.01	4.83	4.34	*
Donor C Test product	0.69	1.42	1.79	0.63	

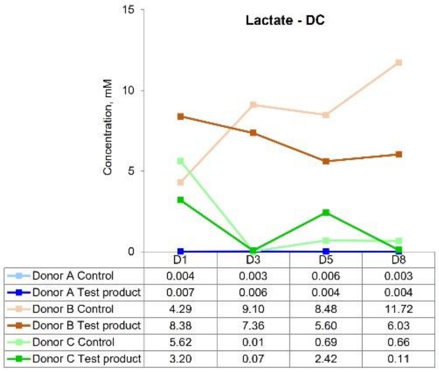

	D1	D3	D5	D8	
Donor A Control	0.004	0.003	0.006	0.003	n.s.
Donor A Test product	0.007	0.006	0.004	0.004	
Donor B Control	4.29	9.10	8.48	11.72	n.s.
Donor B Test product	8.38	7.36	5.60	6.03	
Donor C Control	5.62	0.01	0.69	0.66	n.s.
Donor C Test product	3.20	0.07	2.42	0.11	

Figure S2. Microbial metabolic activity over time (D1, D3, D5, D8) in the proximal (PC) and distal colon (DC) for the SCIME study shown as changes in (A) bSCFA, and (B) ammonium concentrations for individual canine donors (Donors A-C). Each measurement was performed in single repetition. Two-tailed paired t-tests were used to determine significant differences between test product and control. *P <0.05.

bSCFA, branched short chain fatty acid; D, day; DC, distal colon; n.s., not significant; PC, proximal colon; SCIME, Simulator of the Canine Intestinal Microbial Ecosystem

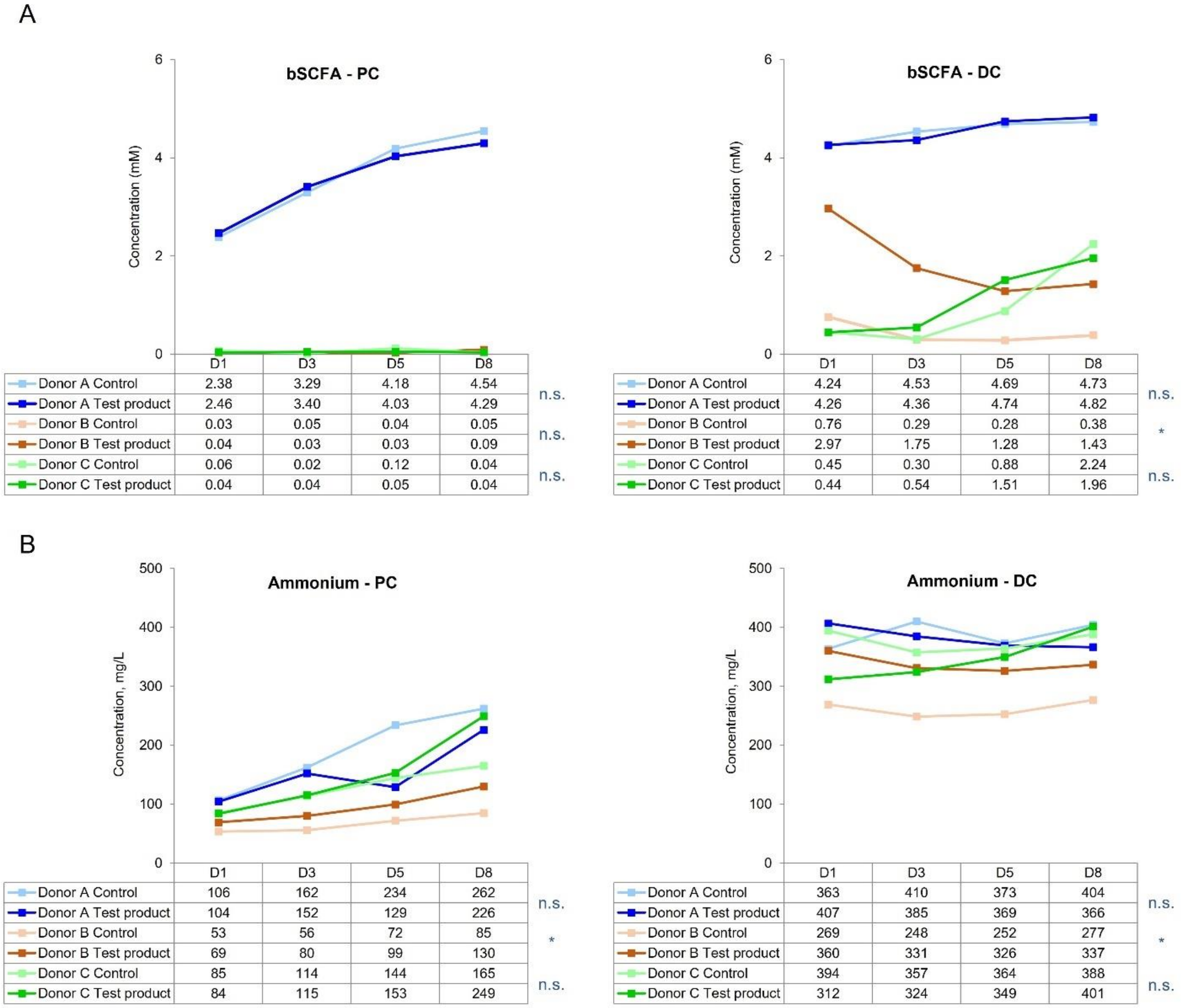

A — bSCFA - PC

	D1	D3	D5	D8	
Donor A Control	2.38	3.29	4.18	4.54	n.s.
Donor A Test product	2.46	3.40	4.03	4.29	
Donor B Control	0.03	0.05	0.04	0.05	n.s.
Donor B Test product	0.04	0.03	0.03	0.09	
Donor C Control	0.06	0.02	0.12	0.04	n.s.
Donor C Test product	0.04	0.04	0.05	0.04	

A — bSCFA - DC

	D1	D3	D5	D8	
Donor A Control	4.24	4.53	4.69	4.73	n.s.
Donor A Test product	4.26	4.36	4.74	4.82	
Donor B Control	0.76	0.29	0.28	0.38	*
Donor B Test product	2.97	1.75	1.28	1.43	
Donor C Control	0.45	0.30	0.88	2.24	n.s.
Donor C Test product	0.44	0.54	1.51	1.96	

B — Ammonium - PC

	D1	D3	D5	D8	
Donor A Control	106	162	234	262	n.s.
Donor A Test product	104	152	129	226	
Donor B Control	53	56	72	85	*
Donor B Test product	69	80	99	130	
Donor C Control	85	114	144	165	n.s.
Donor C Test product	84	115	153	249	

B — Ammonium - DC

	D1	D3	D5	D8	
Donor A Control	363	410	373	404	n.s.
Donor A Test product	407	385	369	366	
Donor B Control	269	248	252	277	*
Donor B Test product	360	331	326	337	
Donor C Control	394	357	364	388	n.s.
Donor C Test product	312	324	349	401	

Table S1: Absolute levels (log cells/mL) of different phyla in the proximal (PC) and distal colon (DC) following treatment with the test product at d1, d3, d5 and d8 of the control/treatment period for donor A, B and C. The intensity of the shading correlates with the absolute abundance, normalized for each of the different phyla (i.e. within each row).

Phylum	PC							
	Donor A							
	Control				Treatment			
	d1	d3	d5	d8	d1	d3	d5	d8
Actinobacteriota	<LOQ	7,32	7,80	7,74	6,92	8,21	8,46	8,54
Bacteroidota	7,27	8,52	8,65	8,50	<LOQ	8,34	8,15	8,72
Firmicutes	8,85	9,35	9,43	9,59	8,85	9,27	9,46	9,51
Proteobacteria	<LOQ	9,12	9,34	9,37	<LOQ	5,81	8,91	9,22

Phylum	PC							
	Donor B							
	Control				Treatment			
	d1	d3	d5	d8	d1	d3	d5	d8
Bacteroidota	<LOQ	<LOQ	6,27	7,36	7,71	8,59	8,94	8,66
Firmicutes	9,25	9,35	9,53	9,47	9,01	9,52	9,55	9,74
Proteobacteria	7,26	5,77	6,29	6,14	6,13	6,37	5,87	6,48

Phylum	PC							
	Donor C							
	Control				Treatment			
	d1	d3	d5	d8	d1	d3	d5	d8
Actinobacteriota	7,23	7,29	8,49	8,52	7,83	7,60	8,38	8,22
Bacteroidota	6,52	6,46	8,07	7,74	7,54	7,53	8,12	5,85
Firmicutes	9,03	9,10	9,55	9,56	9,08	9,37	9,55	9,64
Fusobacteriota	<LOQ	<LOQ	<LOQ	<LOQ	<LOQ	<LOQ	7,12	8,36
Proteobacteria	6,05	<LOQ	7,28	7,24	6,13	6,18	7,05	7,75

Phylum	DC							
	Donor A							
	Control				Treatment			
	d1	d3	d5	d8	d1	d3	d5	d8
Actinobacteriota	6,76	7,59	7,84	7,93	<LOQ	7,61	8,46	8,45
Bacteroidota	7,70	8,29	8,52	8,79	7,87	8,05	8,63	8,82
Cyanobacteria	<LOQ	6,01	<LOQ	<LOQ	<LOQ	<LOQ	<LOQ	<LOQ
Desulfobacterota	<LOQ	<LOQ	<LOQ	<LOQ	<LOQ	<LOQ	5,88	5,84
Firmicutes	8,81	9,23	9,28	9,27	8,79	8,91	9,30	9,32
Fusobacteriota	6,06	6,31	6,63	7,16	6,18	5,78	6,78	6,92
Proteobacteria	7,16	8,95	9,35	9,45	6,73	6,81	8,70	8,95

Phylum	DC							
	Donor B							
	Control				Treatment			
	d1	d3	d5	d8	d1	d3	d5	d8
Actinobacteriota	7,14	7,42	7,63	7,65	7,22	6,83	7,39	7,45
Bacteroidota	8,21	8,88	8,99	8,94	8,62	8,56	9,04	9,13
Firmicutes	9,40	9,51	9,64	9,69	9,24	9,26	9,55	9,60
Fusobacteriota	7,64	<LOQ	<LOQ	<LOQ	8,76	7,64	8,22	8,48
Proteobacteria	7,18	7,05	7,12	7,15	7,69	7,20	7,86	8,07

Phylum	DC							
	Donor C							
	Control				Treatment			
	d1	d3	d5	d8	d1	d3	d5	d8
Actinobacteriota	7,57	7,53	8,22	8,61	7,90	8,29	8,18	8,45
Bacteroidota	8,33	8,26	8,92	8,98	8,49	8,67	8,49	8,84
Firmicutes	9,02	9,18	9,51	9,33	9,08	9,47	9,41	9,54
Fusobacteriota	7,70	7,57	7,94	8,67	8,06	8,70	8,96	8,70
Proteobacteria	6,57	6,47	7,38	7,74	6,63	7,07	7,68	7,98

Table S2: Absolute levels (log cells/mL) of different families in the proximal colon (PC) following treatment with the test product at d1, d3, d5 and d8 of the control/treatment period for donor A, B and C. The intensity of the shading correlates with the absolute abundance, normalized for each of the different phyla (i.e. within each row).

Phylum	Family	PC							
		Donor A							
		Control				Treatment			
		d1	d3	d5	d8	d1	d3	d5	d8
Actinobacteriota	***Bifidobacteriaceae***	<LOQ	7,32	7,80	7,65	6,92	8,21	8,43	8,46
	Coriobacteriaceae	<LOQ	<LOQ	<LOQ	7,01	<LOQ	<LOQ	7,37	7,75
Bacteroidota	***Bacteroidaceae***	<LOQ	<LOQ	7,08	7,13	<LOQ	<LOQ	<LOQ	6,83
	Prevotellaceae	7,27	8,52	8,64	8,48	<LOQ	8,34	8,15	8,71
Firmicutes	***Acidaminococcaceae***	<LOQ	<LOQ	6,19	6,20	<LOQ	<LOQ	<LOQ	<LOQ
	Enterococcaceae	6,13	7,18	7,46	6,31	6,95	6,13	6,27	<LOQ
	Erysipelatoclostridiaceae	<LOQ	6,08	7,89	7,84	6,33	6,30	6,53	6,80
	Erysipelotrichaceae	<LOQ	<LOQ	6,52	6,34	<LOQ	<LOQ	<LOQ	6,32
	Lachnospiraceae	7,58	7,79	8,08	7,72	7,34	6,87	7,52	7,15
	Selenomonadaceae	8,82	9,33	9,39	9,57	8,82	9,27	9,45	9,50
	Veillonellaceae	6,34	6,96	7,52	7,61	6,99	6,93	7,42	7,66
	Veillonellales-Selenomonadales_ unclassified	6,20	6,77	<LOQ	<LOQ	6,59	6,79	7,17	<LOQ
Proteobacteria	***Succinivibrionaceae***	<LOQ	9,12	9,34	9,37	<LOQ	5,81	8,91	9,22
	Sutterellaceae	<LOQ	<LOQ	5,84	6,54	<LOQ	<LOQ	<LOQ	6,09

Phylum	Family	PC							
		Donor B							
		Control				Treatment			
		d1	d3	d5	d8	d1	d3	d5	d8
Bacteroidota	***Bacteroidaceae***	<LOQ	<LOQ	6,27	7,36	<LOQ	<LOQ	<LOQ	7,17
	Prevotellaceae	<LOQ	<LOQ	<LOQ	<LOQ	7,71	8,59	8,94	8,65
Firmicutes	***Clostridiaceae***	6,45	6,30	5,90	6,19	<LOQ	<LOQ	<LOQ	<LOQ
	Erysipelatoclostridiaceae	6,61	6,20	6,06	6,14	<LOQ	6,52	6,20	6,95
	Lachnospiraceae	7,18	5,83	7,35	8,13	<LOQ	7,46	7,98	8,13
	Selenomonadaceae	9,19	9,24	9,43	9,42	8,94	9,50	9,53	9,72
	Streptococcaceae	8,34	8,66	8,81	8,24	8,16	8,21	7,82	7,87
Proteobacteria	***Sutterellaceae***	7,25	<LOQ	6,29	6,05	6,12	6,25	<LOQ	6,20
	Xanthomonadaceae	<LOQ	<LOQ	<LOQ	<LOQ	<LOQ	5,76	<LOQ	6,16

Phylum	Family	PC							
		Donor C							
		Control				Treatment			
		d1	d3	d5	d8	d1	d3	d5	d8
Actinobacteriota	***Coriobacteriaceae***	7,23	7,29	8,49	8,52	7,83	7,60	8,38	8,22
	Eggerthellaceae	<LOQ	<LOQ	<LOQ	6,06	<LOQ	<LOQ	<LOQ	<LOQ
Bacteroidota	***Bacteroidaceae***	6,52	6,46	8,07	7,74	7,54	7,53	8,12	5,85
Firmicutes	***Aerococcaceae***	<LOQ	<LOQ	<LOQ	6,34	<LOQ	<LOQ	<LOQ	6,38
	Clostridiaceae	<LOQ	<LOQ	<LOQ	<LOQ	<LOQ	<LOQ	6,23	5,85
	Lachnospiraceae	6,03	<LOQ	6,83	6,82	6,37	6,26	8,38	8,19
	Lactobacillaceae	7,94	7,94	8,78	8,72	<LOQ	8,37	9,00	9,19
	Selenomonadaceae	8,99	9,05	9,42	9,44	9,07	9,32	9,30	9,38
	Streptococcaceae	<LOQ	<LOQ	<LOQ	<LOQ	7,50	<LOQ	<LOQ	<LOQ
	Veillonellaceae	6,02	7,59	8,53	8,59	<LOQ	7,27	8,52	8,33
	Veillonellales-Selenomonadales_ unclassified	5,99	<LOQ	<LOQ	5,91	5,98	<LOQ	<LOQ	<LOQ
Fusobacteriota	***Fusobacteriaceae***	<LOQ	<LOQ	<LOQ	<LOQ	<LOQ	<LOQ	7,12	8,36
Proteobacteria	***Sutterellaceae***	6,04	<LOQ	7,25	7,21	6,13	6,18	7,04	7,75
	Xanthomonadaceae	<LOQ	<LOQ	5,95	5,91	<LOQ	<LOQ	<LOQ	<LOQ

Table S3: Absolute levels (log cells/mL) of different families in the distal colon (DC) following treatment with the test product at d1, d3, d5 and d8 of the control/treatment period for donor A, B and C. The intensity of the shading correlates with the absolute abundance, normalized for each of the different phyla (i.e. within each row).

Phylum	Family	DC							
		Donor A							
		Control				Treatment			
		d1	d3	d5	d8	d1	d3	d5	d8
Actinobacteriota	*Bifidobacteriaceae*	<LOQ	7,55	7,81	7,87	<LOQ	7,61	8,43	8,36
	Coriobacteriaceae	6,71	6,41	6,70	6,96	<LOQ	<LOQ	7,16	7,75
	Eggerthellaceae	5,76	<LOQ	<LOQ	6,01	<LOQ	<LOQ	5,80	6,14
Bacteroidota	*Bacteroidaceae*	7,64	8,12	8,23	8,37	7,85	7,75	8,43	8,48
	Barnesiellaceae	<LOQ	<LOQ	5,81	6,38	<LOQ	<LOQ	<LOQ	<LOQ
	Prevotellaceae	<LOQ	7,45	7,93	8,38	<LOQ	7,63	7,94	8,13
	Rikenellaceae	<LOQ	6,43	6,11	6,13	<LOQ	<LOQ	6,35	5,97
	Tannerellaceae	6,76	7,55	7,88	8,12	6,56	7,07	7,86	8,35
Cyanobacteria	*Gastranaerophilales_fa*	<LOQ	6,01	<LOQ	<LOQ	<LOQ	<LOQ	<LOQ	<LOQ
Desulfobacterota	*Desulfovibrionaceae*	<LOQ	<LOQ	<LOQ	<LOQ	<LOQ	<LOQ	5,88	5,84
Firmicutes	*Acidaminococcaceae*	<LOQ	5,82	<LOQ	6,13	<LOQ	<LOQ	5,94	6,21
	Clostridia_UCG-014_fa	<LOQ	<LOQ	5,91	<LOQ	<LOQ	<LOQ	<LOQ	<LOQ
	Enterococcaceae	7,16	7,66	8,05	8,33	7,14	7,06	7,71	6,73
	Erysipelatoclostridiaceae	6,05	6,01	6,79	6,93	<LOQ	6,00	6,81	6,41
	Erysipelotrichaceae	6,62	7,11	6,94	6,90	6,44	6,03	6,79	6,57
	Lachnospiraceae	8,08	8,35	8,58	8,25	8,28	8,09	8,62	8,35
	Oscillospiraceae	<LOQ	<LOQ	<LOQ	<LOQ	<LOQ	<LOQ	5,80	<LOQ
	Peptostreptococcaceae	<LOQ	<LOQ	<LOQ	6,44	<LOQ	<LOQ	<LOQ	<LOQ
	Ruminococcaceae	6,36	6,14	6,25	6,41	6,29	6,03	6,51	6,73
	Selenomonadaceae	8,70	9,14	9,14	9,15	8,61	8,82	9,17	9,26
	Veillonellaceae	6,32	7,28	7,35	7,64	6,40	6,76	7,45	7,44
	Veillonellales-Selenomonadales_ unclassified	6,21	<LOQ	<LOQ	<LOQ	6,15	<LOQ	<LOQ	<LOQ
Fusobacteriota	*Fusobacteriaceae*	6,06	6,31	6,63	7,16	6,18	5,78	6,78	6,92
Proteobacteria	*Enterobacteriaceae*	<LOQ	<LOQ	<LOQ	<LOQ	<LOQ	<LOQ	<LOQ	5,84
	Pseudomonadaceae	<LOQ	<LOQ	<LOQ	6,81	<LOQ	<LOQ	6,62	6,60
	Succinivibrionaceae	7,02	8,94	9,34	9,44	5,99	6,11	8,67	8,93
	Sutterellaceae	6,62	7,18	7,58	7,65	6,64	6,71	7,49	7,33

Phylum	Family	DC							
		Donor B							
		Control				Treatment			
		d1	d3	d5	d8	d1	d3	d5	d8
Actinobacteriota	*Coriobacteriaceae*	7,13	7,42	7,63	7,65	7,22	6,83	7,39	7,45
Bacteroidota	*Bacteroidaceae*	8,18	8,88	8,99	8,94	8,59	8,56	9,03	9,12
	Prevotellaceae	7,02	<LOQ	<LOQ	<LOQ	7,43	6,53	6,60	6,87
Firmicutes	*Acidaminococcaceae*	<LOQ	5,76	6,14	6,31	5,81	<LOQ	6,33	6,31
	Clostridiaceae	<LOQ	5,76	5,93	6,58	5,98	<LOQ	<LOQ	<LOQ
	Enterococcaceae	6,18	6,88	7,12	7,43	<LOQ	5,79	<LOQ	5,80
	Erysipelatoclostridiaceae	6,72	6,84	6,31	6,38	6,55	6,27	6,60	6,96
	Erysipelotrichaceae	6,78	6,15	<LOQ	6,38	7,15	6,96	6,68	7,01
	Lachnospiraceae	8,01	7,72	8,22	8,55	8,20	7,79	8,11	7,92
	Peptostreptococcaceae	<LOQ	<LOQ	<LOQ	<LOQ	6,72	7,08	6,78	7,24
	Ruminococcaceae	7,33	<LOQ	<LOQ	<LOQ	7,36	6,28	6,90	6,37
	Selenomonadaceae	9,23	9,25	9,30	9,24	8,95	9,10	9,39	9,50
	Streptococcaceae	8,84	9,14	9,34	9,45	8,81	8,66	8,96	8,87
Fusobacteriota	*Fusobacteriaceae*	7,64	<LOQ	<LOQ	<LOQ	8,76	7,64	8,22	8,48
Proteobacteria	*Alcaligenaceae*	<LOQ	<LOQ	5,93	6,35	<LOQ	<LOQ	<LOQ	<LOQ
	Enterobacteriaceae	<LOQ	<LOQ	<LOQ	<LOQ	7,32	6,85	7,41	7,64
	Pseudomonadaceae	<LOQ	<LOQ	<LOQ	<LOQ	<LOQ	6,45	7,17	7,79
	Sutterellaceae	7,18	7,04	7,06	6,91	7,43	6,77	7,51	7,13
	Xanthomonadaceae	<LOQ	<LOQ	6,01	6,53	<LOQ	<LOQ	<LOQ	<LOQ

Phylum	Family	DC							
		Donor C							
		Control				Treatment			
		d1	d3	d5	d8	d1	d3	d5	d8
Actinobacteriota	*Bifidobacteriaceae*	6,88	7,23	7,89	8,08	7,18	7,77	7,82	7,95
	Coriobacteriaceae	7,47	7,21	7,94	8,46	7,81	8,13	7,94	8,29
	Eggerthellaceae	<LOQ	<LOQ	5,75	5,86	<LOQ	<LOQ	<LOQ	<LOQ
Bacteroidota	*Bacteroidaceae*	8,33	8,26	8,92	8,98	8,49	8,67	8,49	8,84
	Barnesiellaceae	<LOQ	<LOQ	6,53	6,51	<LOQ	<LOQ	<LOQ	<LOQ
Firmicutes	*Aerococcaceae*	<LOQ	<LOQ	<LOQ	5,96	<LOQ	<LOQ	<LOQ	6,13
	Clostridia_UCG-014_fa	<LOQ	<LOQ	5,75	6,49	<LOQ	<LOQ	<LOQ	6,07
	Clostridiaceae	<LOQ	<LOQ	<LOQ	<LOQ	<LOQ	<LOQ	5,84	<LOQ
	Enterococcaceae	<LOQ	<LOQ	6,05	<LOQ	<LOQ	<LOQ	<LOQ	<LOQ
	Erysipelotrichaceae	<LOQ	<LOQ	5,75	6,66	<LOQ	<LOQ	<LOQ	6,88
	Lachnospiraceae	8,11	8,04	8,51	8,40	8,24	8,21	8,22	8,57
	Lactobacillaceae	7,53	7,87	8,45	8,81	<LOQ	8,16	8,92	9,20
	Oscillospiraceae	<LOQ	<LOQ	5,75	6,37	<LOQ	<LOQ	5,92	6,18
	Peptostreptococcaceae	<LOQ	<LOQ	6,98	7,64	5,88	6,38	7,38	7,34
	Ruminococcaceae	6,69	<LOQ	<LOQ	5,96	7,23	<LOQ	<LOQ	6,50
	Selenomonadaceae	8,94	9,11	9,37	9,03	8,99	9,39	9,15	9,02
	Streptococcaceae	6,26	<LOQ	<LOQ	<LOQ	7,35	8,12	5,92	<LOQ
	Veillonellaceae	6,24	7,63	8,44	8,15	<LOQ	7,58	8,11	8,60
	Veillonellales-Selenomonadales_ unclassified	6,14	<LOQ	<LOQ	<LOQ	<LOQ	<LOQ	<LOQ	<LOQ
Fusobacteriota	*Fusobacteriaceae*	7,70	7,57	7,94	8,67	8,06	8,70	8,96	8,70
Proteobacteria	*Enterobacteriaceae*	<LOQ	<LOQ	<LOQ	<LOQ	<LOQ	6,06	<LOQ	<LOQ
	Pseudomonadaceae	<LOQ	<LOQ	6,86	6,91	<LOQ	6,06	7,36	7,36
	Sutterellaceae	6,52	6,41	7,19	7,67	6,62	6,97	7,39	7,86
	Xanthomonadaceae	<LOQ	<LOQ	6,05	<LOQ	<LOQ	<LOQ	<LOQ	<LOQ
